# Supplementary material for: Exposure to Hantavirus is a Risk Factor Associated with Kidney Diseases in Sri Lanka: A Cross Sectional Study
Source: Viruses. 2019 Jul 31;11(8):700. doi: 10.3390/v11080700 (PMC6723923; doi:10.3390/v11080700)

Supplementary data files

Exposure to hantavirus is a risk factor associated with kidney diseases in Sri Lanka: A cross sectional study

by Yomani D. Sarathkumara, Chandika D. Gamage, Sithumini Wimalasiri, Devinda S. Muthusinghe, Nishantha Nanayakkara, Lishanthe Gunarathne, Kenta Shimizu, Yoshimi Tsuda, and Jiro Arikawa, and Kumiko Yoshimatsu

**Contents**	**Page**
Supplementary Materials 1. Determination of sample size	2
Supplementary Materials 2. Questionnaire in English and Consent form to recruit participant	4
Supplementary Materials 3. Binary logistic regression variables included in the model that predicted the probability of an individual being seropositive to THAIV	13
Supplementary Materials 4. Results of univariate descriptive analysis<br>(1) Univariate descriptive analysis of renal patients from KTH<br>(2) Univariate descriptive analysis of controls from Kandy district<br>(3) Univariate descriptive analysis of renal patients from GK DH<br>(4) Univariate descriptive analysis of controls from Girandrukotte	14
Supplementary Materials 5. Complete results for comparison of possible risk factors for exposure to hantavirus in Kandy and Girandurukotte	19
Supplementary Materials 6. IFA profiles of THAIV infected and rNP of THAIV expressing in Vero E6 cells	22
Supplementary Materials 7. ELISA results of seropositive and seronegative sera groups	23

Supplementary Materials 1. Determination of sample size

Hospital based: Sample size from Girandurukotte district hospital was estimated considering CKDu prevalence as 15% that has been reported elsewhere and the Girandurukotte district hospital (Rajapakse et al., 2016). Sample size from Teaching Hospital Kandy was decided considering the approximate registered number of patients in the renal clinic conducted (Personal communication).
Estimated sample size for Girandurukotte district hospital n = 196
Estimated sample size for Teaching Hospital Kandy size: n = 100

Community based: Medical Office of Health (MOH) areas were randomly selected from Kandy district. Regional Director of Health Services (RDHS), Kandy and Badulla granted permission for collecting blood samples and demographic data from the community in the selected MOH/PHI areas. People who are 18 years old and above, with absence of past history or currently diagnosed as a patient of kidney disease, reside in Kandy and Girandurukotte areas who are voluntarily given a blood sample were recruited to the study through public health inspectors (PHIs) attached to selected MOH areas from the respective districts.
Sample size was calculated according to following formula;

$$n = \frac{Z^2 P(1-P)}{d^2}$$

Where n is the sample size, Z is the statistic corresponding to level of confidence, P is expected prevalence (that can be obtained from same studies or a pilot study conducted by the researchers), and d is precision (corresponding to effect size). The level of confidence (CI) considered being 95%. People Sample size was calculated considering the population statistics of the particular district (Department of Census and Statistics, Sri Lanka).

<u>Sample size for Kandy district:</u>
Total number of population in Kandy district - 1,279,028
Assumptions:
Precision = 5.00 %
Prevalence = Unknown (Thus use 50.00 % for calculation)
Population size = 1279028
95% Confidence Interval specified limits [45% -- 55%]
(these limits equal prevalence plus or minus precision)

Estimated sample size: n = 385

Sample size for Badulla district:

Total number of population in Badulla district - 779,983

Assumptions:

Precision = 5.00 %

Prevalence = Unknown (Thus use 50.00 % for calculation)

Population size = 779983

95% Confidence Interval specified limits [45% -- 55%]

(these limits equal prevalence plus or minus precision)

Estimated sample size: n = 384

Supplementary Materials 2. Consent form to recruit participant and Questionnaire in English

Consent form to recruit participants

I am Dr. Chandika Gamage working in the Department of Microbiology, Faculty of Medicine, University of Peradeniya

This research is conducted to measure the prevalence for past exposure of leptospirosis and hantaviruse infection in selected areas among clinically diagnosed chronic kidney disease with uncertain etiology (CKDu) patients and community people reside in the respective areas.

I am going to give you information and invite you to be part of this research. You do not have to decide today whether or not you will participate in the research. Before you decide, you can talk to anyone you feel comfortable with about the research.

There may be some words that you do not understand. Please ask me to stop as we go through the information and I will take time to explain. If you have questions later, you can ask them of me, the study doctor or the staff.

Part 1

Information sheet:
Title of the research: Exposure to Leptospirosis and Hantavirus Infection in a selected cohort of chronic kidney disease with uncertain etiology (CKDu) patients and non-CKDu individuals in selected CKDu affected areas of Sri Lanka

Version Number: 1.1, September 2014
Date: 07/09/2016

Purpose of the research
Purpose of the research is measure the prevalence of leptospirosis and hantavirus infection of the area, as above diseases are endemic and emerging zoonotic diseases in the country respectively which can cause acute kidney damage which may progress into chronic kidney disease.
The information we tabulate will lead you to know whether you have been previously infected with these mentioned infectious diseases. Further, this information will be able to describe

seroepidemiology of leptospirosis and hantavirus infection and to know it's role as a possible risk factor causing CKDu.

Type of Research:

The participants should be permanent residents of selected area who are 18 years old. The data will be collected by a questionnaire provided and by collecting blood by venepuncture method by well-trained health care personnel.

Participant selection:

Your participation in this research is entirely voluntary. It is your choice whether to participate or not. The participants are selected randomly with an age of 18 years old and above. You may change your mind later and stop participating even if you agreed earlier. Resigning from the research will not be affected on your medical care in any way.

Procedures and Protocol

First, we will give you the inform consent form to read and you have the opportunity to ask any questions and discuss about the study.

Once you read and understand the information you can give your consent by signing the consent certificate.

You will be asked to fill out a structured questionnaire and it will be take approximately 10-15 minutes.

Then, well-trained personnel in the team will collect blood only one time by venipuncture, i.e.; pierce a vein in the elbow area and collect about 5ml of blood by using sterilized needles and syringes to an appropriate container.

The blood samples will be properly transported to the lab in the Microbiology Department, Faculty of Medicine, University of Peradeniya, and there, the specimen will be processed and will analyse for antibodies for leptospirosis and hantavirus infection.

The collected blood will be only used for the test mentioned above and will be stored until the data analysis.

Duration:

The research takes place over 12 months in total. Your participation will be needed once only and there's no need of traveling to any medical facility or institute regarding this research further.

Risks:

Drawing blood for serological and molecular investigations has a minimal risk. We will ensure that all possible preventive measures according to the current standards and guidelines will be strictly adhered to prevent any risks of blood drawing. All personnel will be trained to detect and handle complications during blood drawing and will be provided with medical assistance when needed.

Benefits:

If you participate in this research, you will have the following benefits:

You will get to know whether you have been infected with either of the diseases mentioned above. The information gathered from this study will be beneficial to enhance medical interventions and prevention of diseases in the areas.

Confidentiality:

The information that we collect from this research project will be kept confidential. Information about you that will be collected during the research will be put away and no-one but the researchers will be able to see it. Any information about you will have a number on it instead of your name. Only the researchers will know what your number is and we will lock that information up with a lock and key. It will not be shared with or given to anyone.

Right to Refuse or Withdraw:

You do not have to take part in this research if you do not wish to do so. You may also stop participating in the research at any time you choose. It is still your choice and all of your rights will still be respected.

Who to Contact:

If you have any questions you may ask them now or later, even after the study has started. If you wish to ask questions later, you may contact any of the following:

Name: Dr. Chandika Gamage (Principal Investigator)
Address: Department of Microbiology, Faculty of Medicine, University of Peradeniya
Telephone number/e-mail: +94771661460
gamagecd@gmail.com / chandika@pdn.ac.lk

Name: Dr. Nishantha Nanayakkara
Telephone number/e-mail: +94812314391
nishansrikandy@yahoo.co.in

PART II

Certificate of Consent:

I have read the foregoing information, or it has been read to me. I have had the opportunity to ask questions about it and any questions that I have asked have been answered to my satisfaction. I consent voluntarily to participate as a participant in this research.

Name of Participant: ____________________________________

Signature of Participant: _________________

Date: _________________

Day/month/year

If illiterate:

A literate witness must sign (if possible, this person should be selected by the participant and should have no connection to the research team). Participants who are illiterate should include their thumb-print as well.

I have witnessed the accurate reading of the consent form to the potential participant, and the individual has had the opportunity to ask questions. I confirm that the individual has given consent freely.

Print name of witness: __________________________________

AND Thumb print of participant: _________________

Signature of witness: _________________

Date: _________________

Day/month/year

Questionnaire in English

Laboratory Number:

Hantavirus Infection Survey

Interview Date : _________ (DD/MM/YY) NIC Number : ___________

Clinic Number : _________

Province : _____________ District : ___________

Divisional secretariat : ___________

Respondent Demographics

1. Respondent's name	
2. Age (Years)	
3. Please select respondents gender	0 = Male<br>1 = Female
4. What is the highest level of school that you have attended?	1 = Primary<br>2 = Middle<br>3 = Secondary<br>4 = Higher-secondary<br>5 = University<br>6 = Other, please specify:
5. Address	
6. Contact number	
7. Residence in the same geographical area (last 5 years)	1 = Yes 0 = No

Family and Past Medical History

8. In what village were you born?	Village :________________________<br>Province :_______________________
9. Have you shifted from the village you were born?	1 = Yes 0 = No
10. If yes to previous question, in what are other locations have you resided?	Village: Province:<br>1)<br>2)<br>3)
11. Are you married?	1 = Yes 0 = No
12. What is your primary occupation?	1 = rice farmer<br>2 = chena farmer<br>3 = other, please specify
13. How long have you had this occupation?	1 = 1-3 years<br>2 = 4-7 years<br>3 = 8-10 years<br>4 = >10 years
14. Have you ever been diagnosed with or experienced the following? (please circle all those that apply)	1 = Diabetes<br>2 = Hypertension<br>3 = Kidney disease<br>4 = Respiratory disease (asthma, hypersensitive to allergens etc)<br>5 = Venomous snake bite (requiring treatment)

15. Have you ever been diagnosed with Leptospirosis?	1 = Yes 0 = No		
16. If yes, have you been prescribed with any antibiotics?	1 = Yes 0 = No		
17. Are you currently being treated for any other illness/disease requiring regular medical visits?	1 = Yes 0 = No		
18. If yes to previous question, please list	1)<br>2)<br>3)		
19. Has anyone in your family been diagnosed with CKD/CKDu?	1 = Yes 0 = No		
20. If yes, please list relationship to you.	Relation:		
21. Have your parents ever been diagnosed with the following? (please circle all those that apply)	Diagnosis:<br>1) Diabetes<br>2) Kidney Disease<br>3) Hypertension	<u>Mother</u><br>Yes / No<br>Yes / No<br>Yes / No	<u>Father</u><br>Yes / No<br>Yes / No<br>Yes / No
22. Did they undergo treatment for illness?	1 = Yes 0 = No		
23. Do you have children?	1 = Yes 0 = No		
24. Have your children ever been diagnosed with the following? (please circle all those that apply)	Diagnosis:<br>1) Diabetes<br>2) Kidney Disease<br>3) Hypertension<br><br>1 = Yes 0 = No	<u>Son</u>	<u>Daughter</u>
25. Did they undergo treatment for illness?	1 = Yes 0 = No		
26. Have any neighbors been diagnosed with CKD/CKDu that you know of?	1 = Yes 0 = No		

<u>Agricultural Information</u>

27. Do you own or tend land used for farming (agriculture, chena farming, home garden)	1 = Yes 0 = No
28. If you farm, where do you farm?	1 = Lowlands<br>2 = Highlands<br>3 = Both<br>4 = Other, specify
29. Which farming activities do you participate?	*Please check all that apply:*<br>___ Land preparation<br>___ Fertilizer and pesticide application<br>___ Harvesting
30. Do you use any of the following chemicals to protect crops?	Circle all that apply:<br>1 = Insecticides<br>2 = Herbicides<br>3 = Pesticides<br>4 = Fungicides<br>5 = Fertilizer
31. What protective equipment do you use during farming activities?	Circle all that apply:<br>1 = Hand protection<br>2 = Eye protection<br>3 = Leg protection<br>4 = Respiratory protection<br>5 = Other, please specify

32. Do you store rice/ crops?	1 = Yes 0 = No
33. If Yes, Where do you store them?	0 = Inside the house<br>1 = Outside the house
34. How often do you visit the store room?	1 = Daily<br>2 = Once or twice per week<br>3 = Once or twice per month
35. On average how long do you spend in store room (in minutes)	1= < 5 minutes<br>2= > 5 minutes but < 15 minutes<br>3 = > 15 minutes
36. Do you have windows in the rice/crop storage place to allow air circulation?	1 = Yes 0 = No
37. Do you wear any respiratory protection during stay in the store room	1 = Yes 0 = No
38. Are there any traditional practices that were used during storing the crops that are no longer practiced? (eg: Vee bissa)	
39. Have you ever noticed rats in the rice/crop storage place?	1 = Yes 0 = No
40. If Yes, Have you ever noticed dried rodent faeces?	0 = No<br>1 = Yes<br>2 = Do not know

Animal Exposure/ Health

41. Do you make any contacts with animals (as a pet)<br>*Circle all that apply	1 = Dog<br>2 = Cat<br>3 = Other, specify<br>4 = NA	
42. Have you seen any rats inside or surroundings of your house?	1 = Yes 0 = No	
43. If Yes, How often do you see rats?	1 = Daily<br>2 = Once or twice per week<br>3 = Once or twice per month	
44. Do you have or keep any livestock animals?	1 = Yes 0 = No	
45. Have you ever noticed dried rodent faeces?	0 = No<br>1 = Yes<br>2 = Do not know	
46. Have you noticed any ill animals in the past month?<br>***Please list the ill animals observed, Circle [NA] if no animals have been observed as ill***	Pet [NA]<br>1)<br>2)<br>3)	Livestock [NA]<br>1)<br>2)<br>3)
47. What are your sources of water used for livestock?	1 = Tube Well<br>2 = Dug Well<br>3 = River<br>4 = Tank/Reservoir<br>5 = Rain Water Harvest<br>6 = Other, specify<br>7 = NA	
48. Do see wild animals surrounding your residence?	0 = No<br>1 = Yes<br>2 = Do not know	

Water and Sanitation

49. What are your sources of water used for drinking and cooking? (circle all that apply)	Drinking:<br>1 = Tube Well<br>2 = Dug Well<br>3 = River<br>4 = Tank/Reservoir<br>5 = Bottled water<br>6 = Rain water harvest<br>7 = Other, specify	Cooking:<br>1 = Tube Well<br>2 = Dug Well<br>3 = River<br>4 = Tank/Reservoir<br>5 = Bottled water<br>6 = Rain water harvest<br>7 = Other, specify
50. Do you treat your drinking water (boiling, filter, etc.)?	1 = Yes 0 = No	

****If the respondent is a CKD/CKDu patient please fill the following details;**

51. When did you clinically diagnose as CKD/CKDu?<br>***Mention the year or how many years back***	
52. What is the diagnosed CKD/CKDu stage?	1 = Stage 1<br>2 = Stage 2<br>3 = Stage 3<br>4 = Stage 4<br>5 = Stage 5<br>6 = ESRD<br>7 = Do not know<br>* *ESRD-End-stage kidney disease*
53. If the patient is clinically diagnosed as CKD or CKDu?	0 = Chronic Kidney Disease (CKD)<br>1 = Chronic Kidney Disease of Uncertain etiology (CKDu)<br>2 = Do not know
54. Do you undergo treatments?<br>***If Yes, mention the duration (in months or years)***	1 = Yes Duration : ___________<br>0 = No

Supplementary Materials 3. Binary logistic regression variables included in the model that predicted the probability of an individual being seropositive to THAIV

Variable	Variable description	Nominal reference level
Exposure	Binary response variable, individuals with THAIV-specific IgG antibodies are denoted "P" (Positive) and sero-negative individuals "N" (Negative)	
Status	Dichotomous independent variable: Renal patients or Control	Renal Patient
Age	Dichotomous independent variable: age ≥ 40 years denoted as "1" and age < 40 years denoted as "0"	Age ≥ 40 years
Gender	Dichotomous independent variable: female or male	Male (M)
Occupation	Dichotomous independent variable: Farmer or non-farmer	Farmer
Engaged in agriculture related activities	Dichotomous independent variable: Yes or No	Yes
Store crops at house	Dichotomous independent variable: Yes or No	Yes
Sighting rodent at home or surrounding	Dichotomous independent variable: Yes or No	Yes
Presence of rodent excreta at home or surrounding	Dichotomous independent variable: Yes or No	Yes

Supplementary Materials 4. Univariate descriptive analysis

(1) Renal patients in Kandy: Nine out of the 50 renal patients (18%) were positive for anti-hantavirus IgG antibodies (Table 1). These 9 renal patients consisted of 7 CKD patients, an end-stage renal disease (ESRD) patient and an acute kidney injury (AKI) patient. Furthermore, 88.9% (8/9) of the seropositive patients were 40 years or o older. There was a predominance of men among those who were seropositive, with 7 (77.8%) males and only 2 (22.2%) females identified; however, this difference was not significant (Supplementary Appendix 5). Known causes for developing CKD, such as diabetes mellitus, hypertension and snake bites, were also evaluated. However, there was no significant difference between the presence or absence of these known risk factors for CKD and exposure to hantavirus among renal patients in Kandy. There were 3 paddy farmers in this study group; interestingly, all 3 farmers had seroconverted. Exposure to hantavirus was found to be significantly more common among individuals who engaged in agricultural activities than among those who did not perform agricultural work (χ^2 =4.98, p < 0.05). However, storing crops or rice at home and the presence of rats and their excreta in crop stores were not found to be risk factors for acquiring hantavirus infections. In total, 88.9% of the seropositive patients reported observing living rodents at their home or in the surrounding area.

**Kandy Teaching Hospital Patients**	**Total (n=50)**		**Exposed to hantavirus (n=9)**		**χ2**	***P* value**
	**n**	**%**	**n**	**%**		
1. Age						
40 years or above	46	92.0	8	88.9	0.14	0.560
Less than 40 years	4	8.0	1	11.1		
2. Gender						
Male	28	56.0	7	77.8	2.11	0.266
Female	22	44.0	2	22.2		
3. Occupation						
Farming	3	6.0	3	33.3	14.54	
Non-farming	47	94.0	6	66.7		
4. Risk factors for CKD						
Presence	42	84.0	6	66.7	2.45	0.143
Absence	8	16.0	3	33.3		
5. Exposure to agriculture						
Yes	13	26.0	5	55.6	4.98	**0.039**
No	37	74.0	4	44.4		
6. Storing crop at house						
Yes	6	12.0	2	22.2	1.09	0.292
No	44	88.0	7	77.8		
7. Rats seen at home or surroundings						
Yes	33	66.0	8	88.9	2.56	0.14
No	17	34.0	1	11.1		
8. Dried rodent faeces seen at home or surrounding						

Yes	17	34.0	4	44.4	0.53	0.467
No	33	66.0	5	55.6		

(2) Controls in Kandy

As shown in Fig. 1B, 290 blood samples were obtained from 6 randomly selected PHI areas in the Kandy district. A total of 270 individuals with normal serum creatinine levels (0.5–1.2 mg/dL) were recruited as controls. The prevalence of exposure to hantavirus was 7% (19/270) among the controls from the Kandy district. Among the controls, the seroprevalence was higher in those 40 years or older (73.7%) when compared to those less than 40 years old (26.3%). The seroprevalence was almost identical in both genders in the total control population in Kandy (Supplementary Appendix 6). Furthermore, 12 out of the 19 hantavirus-exposed people had seen or encountered rodents, although the difference between those who had and had not observed rodents among the seropositive controls was not significant.

**Kandy Controls**	**Total (n=270)**		**Exposed to hantavirus (n=19)**		**χ2**	***P* value**
	**n**	**%**	**n**	**%**		
1. Age						
40 years or above	180	66.7	14	73.7	0.45	0.501
Less than 40 years	90	33.3	5	26.3		
2. Gender						
Male	126	46.7	10	52.6	0.29	0.589
Female	144	53.3	9	47.4		
3. Occupation						
Farming	13	4.8	1	5.3	0.01	1
Non-Farming	257	95.2	18	94.7		
4. Risk factors for CKD						
Presence	59	21.9	3	15.8	0.44	0.773
Absence	211	78.1	16	84.2		
5. Agriculture information						
Yes	56	20.7	7	36.8	3.22	0.073
No	214	79.3	12	63.2		
6. Storing crop at house						
Yes	30	11.1	4	21.1	2.05	0.244
No	240	88.9	15	78.9		
7. Rats seen at home or surroundings						
Yes	206	76.3	12	63.2	1.95	0.163
No	64	23.7	7	36.8		
8. Dried rodent faeces seen at home or surrounding						
Yes	108	40.0	7	36.8	0.09	0.771
No	162	60.0	12	63.2		

(3) Renal patients in Girandurukotte

As shown in Table 1, a high seroprevalence of hantavirus antibodies, 50% (52/104), was detected among the renal patients in Girandurukotte. The majority of the seroconverted individuals were 40 years or older (92.3%). The presence of known risk factors for CKD did not show any association with exposure to the virus. Higher seroprevalence was observed in men (80.8%) than in women (19.2%), and the difference was statistically significant in the seroconverted group of renal patients ($\chi2 = 8.57$, $p < 0.05$). Among the 104 renal patients, 92 were paddy farmers and 47 were seropositive for THAIV. The close contact of rodents with humans increases the likelihood of exposure to the excreta of infected rodents. Despite direct exposure to rodents and their excreta, no statistically significant difference was found in the seroconverted group (Supplementary Appendix 7).

**Girandrukotte District Hospital Patients**	**Total (n=104)**		**Exposed to hantavirus (n=52)**		**$\chi2$**	***P value***
	**n**	**%**	**n**	**%**		
1. Age						
40 years or above	95	91.3	48	92.3	0.122	1
Less than 40 years	9	8.7	4	7.7		
2. Gender						
Male	70	67.3	42	80.8	8.57	**0.003**
Female	34	32.7	10	19.2		
3. Occupation						
Farming	92	88.5	47	90.4	0.38	0.539
Non-Farming	12	11.5	5	9.6		
4. Risk factors for CKD						
Presence	67	64.4	30	57.7	2.06	0.152
Absence	37	35.6	22	42.3		
5. Agriculture information						
Farming	98	94.2	50	96.2	0.71	0.400
Non-farming	6	5.8	2	3.8		
6. Storing crop at house						
Yes	97	93.3	48	92.3	0.15	0.696
No	7	6.7	4	7.7		
7. Rats seen at home or surroundings						
Yes	84	80.8	45	86.5	2.23	0.135
No	20	19.2	7	13.5		

8. Dried rodent faeces seen at home or surrounding						
Yes	72	69.2	37	71.2	0.18	0.671
No	32	30.8	15	28.8		

(4) Controls in Girandurukotte

In total, 264 serum samples were obtained from community members residing in the Girandurukotte area. Among them, 242 individuals with normal serum creatinine levels (0.5–1.2 mg/dL) were recruited as controls for this study. In this group, 42 (17.4%) controls were positive for hantavirus antibodies. Among the seroconverted individuals, 76.2% were found to be 40 years or older. Among the controls, exposure to the virus and age 40 years or older were found to have a significant association ($\chi2 = 4.84$, $p < 0.05$) (Supplementary Appendix 8). Men were more commonly seropositive than women among the controls, with a significant association of male gender with exposure to hantavirus ($\chi2 = 4.64$, $p < 0.05$). Known risk factors for CKD, occupation, exposure to agricultural activities and storing crops at home were not associated with the risk of exposure. We found no evidence of direct contact with rodents and their excreta, and no significant association was detected.

**Girandurukotte Controls**	**Total (n=242)**		**Exposed to hantavirus (n=42)**		**χ2**	***P* value**
	**N**	**%**	**n**	**%**		
1. Age						
40 years or above	148	61.2	32	76.2	4.84	**0.028**
Less than 40 years	94	38.8	10	23.8		
2. Gender						
Male	98	40.5	25	59.5	4.64	**0.006**
Female	144	59.5	17	40.5		
3. Occupation						
Farming	112	46.3	25	59.5	3.59	0.058
Non-Farming	130	53.7	17	40.5		
4. Risk factors for CKD						
Presence	52	21.5	11	26.2		0.414
Absence	190	78.5	31	73.8	0.67	
5. Agriculture information						
Farming	179	74.0	35	83.3	2.32	0.128
Non-farming	63	26.0	7	16.7		
6. Storing crop at house						

Yes	159	65.7	28	66.7	0.02	0.885
No	83	34.3	14	33.3		
7. Rats seen at home or surroundings						
Yes	197	81.4	34	81.0	0.01	0.934
No	45	18.6	8	19.0		
8. Dried rodent faeces seen at home or surrounding						
Yes	137	56.6	21	50.0	0.90	0.342
No	105	43.4	21	50.0		

Supplementary Materials 5. Comparison of possible risk factors for exposure to hantavirus in Kandy and Girandurukotte

Category	Kandy				Girandurukotte			
	Anti-hantavirus antibody		$\chi 2$	*P* value	Anti-hantavirus antibody		$\chi 2$	*P* value
	Positive	Negative			Positive	Negative		
**1. Age**								
Age category of ≥ 40 years								
Renal patients	8	38	3.85	**0.050**	48	47	21.89	**<0.001**
Control	14	166			32	116		
Age category of < 40 years								
Renal patients	1	3	2.42	0.235	4	5	7.99	**0.005**
Control	5	85			10	84		
**2. Gender**								
Female								
Renal patients	2	20	0.25	0.642	10	24	6.53	**0.011**
Community	9	135			17	126		
Male								
Renal patients	7	21	6.79	**0.010**	42	28	20.3	**<0.001**
Community	10	116			25	73		
**3. Occupation**								
Paddy farming								

Continued

Renal patients	3	0	11.08	**0.007**	47	45	18.3	**<0.001**
Community	1	12			25	87		
Other occupations								
Renal patients	6	41	1.81	0.178	5	7	18.3	**0.009**
Community	18	239			17	113		
**4. Exposure to agriculture**								
Yes								
Renal patients	5	8	4.95	**0.030**	50	48	29.48	**<0.001**
Community	7	49			35	144		
No								
Renal patients	4	33	1.43	0.232	2	4	2.32	0.170
Community	12	202			7	55		
**5. Storing crop at house**								
Yes								
Renal patients	2	4	1.44	0.260	48	49	29.32	**<0.001**
Community	4	26			28	131		
No								
Renal patients	7	37	4.85	**0.028**	4	3	6.55	**0.027**
Community	15	225			14	69		
**6. Rats seen at home or surroundings**								
Yes								
Renal patients	8	25	12.58	**<0.001**	45	39	38.42	**<0.001**

Community	12	194			34	163		
No								
Renal patients	1	16			7	13		
			0.39	0.535			2.31	0.128
Community	7	57			8	37		
**7. Dried rodent feces seen at home or surrounding**								
Yes								
Renal patients	4	13			37	35	30.61	**<0.001**
			5.32	**0.040**				
Community	7	101			21	116		
No								
Renal patients	5	28			15	17		
			2.07	0.151			9.14	**0.002**
Community	12	150			21	84		

Supplementary Materials 6. IFA profiles of THAIV infected and rNP of THAIV expressing in Vero E6 cells

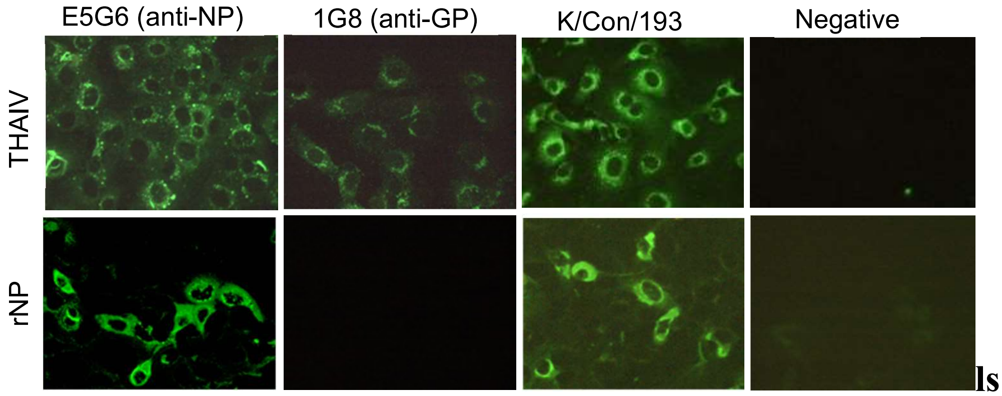

Supplementary Materials 7. ELISA results of seropositive and seronegative sera groups

Positive group (n=61) and negative group (n=42) of sera were applied to ELISA by using HS103 which was truncated NP of HTNV N-terminal 103 amino acids expressed by *E. coli* vector (a) and rNP of SEOV expressed in High Five cells by baculovirus vector (b) as antigens.

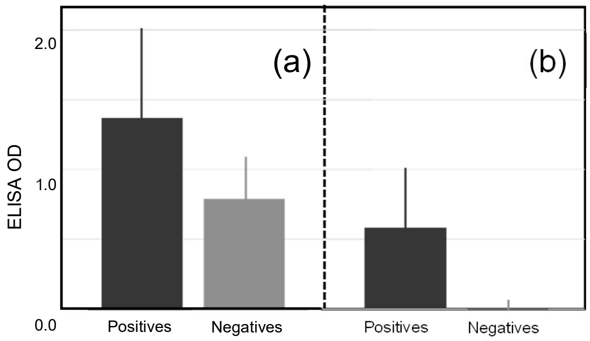

Supplement: Supplementary file 1 [file viruses-11-00700-s001.pdf]
